# Effective dispersal and density-dependence in mesophotic macroalgal forests: Insights from the Mediterranean species *Cystoseira zosteroides*

**DOI:** 10.1371/journal.pone.0191346

**Published:** 2018-01-12

**Authors:** Pol Capdevila, Cristina Linares, Eneko Aspillaga, Joan Lluís Riera, Bernat Hereu

**Affiliations:** Departament de Biologia Evolutiva, Ecologia i Ciències Ambientals, Facultat de Biologia, Universitat de Barcelona, Barcelona, Spain; The University of Hong Kong, HONG KONG

## Abstract

Dispersal and recruitment are fundamental processes for population recovery following disturbances in sessile species. While both processes are well understood for many terrestrial species, they still remain poorly resolved for some macroalgal species. Here we experimentally investigated the effective dispersal and recruit survival of a mesophotic Mediterranean fucoid, *Cystoseira zosteroides*. In three isolated populations, four sets of settlement collectors were placed at increasing distances (from 0 to 10 m) and different orientations (North, South, East and West). We observed that effective dispersal was restricted to populations’ vicinity, with an average of 6.43 m and not further than 13.33 m, following a Weibull distribution. During their first year of life, survival was up to 50%, but it was lower underneath the adult canopy, suggesting a negative density-dependence. To put our results in a broader context we compared the effective dispersal of other fucoid and kelp species reported in the literature, which confirmed the low dispersal ability of brown algae, in particular for fucoids, with an effective dispersal of few meters. Given the importance of recruitment for the persistence and recovery of populations after disturbances, these results underline the vulnerability of *C*. *zosteroides* and other fucoid species to escalating threats.

## Introduction

The increase of anthropogenic stressors (e.g. coastal development, overexploitation, pollution) has driven the loss of key habitat-forming organisms, such as terrestrial plants, corals, and algae, rendering their populations more fragmented, isolated and vulnerable to further sources of disturbance [1–4]. In this context, dispersal and recruitment play a crucial role in maintaining the resilience and ensuring the long-term population stability of habitat-forming species [1–3]. At the local scale, dispersal and successful recruitment determine population dynamics and structure, also driving their recovery after disturbances; while at larger scales these processes ensure population connectivity, gene flow and the colonization of new locations [4–6]. Thus, understanding the dispersal of these species may be crucial to predicting their ability to respond to local and global stressors, as well as to assess the scale at which management strategies may be effective [7–9].

Nonetheless, the wealth of dispersal ecology studies in terrestrial plants [10,11] contrasts with the relative poor mechanistic understanding about the dispersal and settlement of other habitat-forming species like canopy-forming macroalgae [12–15]. Large brown algae of the orders Laminariales (kelps) and Fucales (fucoids) play a key ecological role as habitat-forming species in temperate marine ecosystems worldwide [16–18]. Their dispersal is likely a very passive process, as it occurs through the release of non-motile (or with limited mobility) propagules (spores or zygotes) in the water column [19,20]. Consequently, macroalgae are located at the lowest end of the range of dispersal distances reported for the marine realm [8,21,22]. Still, their propagule stages are microscopic and elusive to study, so their dispersal scales remain poorly understood for many species.

In the Mediterranean Sea, *Cystoseira* spp. are late successional species which conform important forest-like assemblages from the intertidal to sublittoral zone (in some cases deeper than 50 m), providing food and shelter for many associated organisms, enhancing local biodiversity [18,23]. During the last decades, a widespread decline of these assemblages has been documented in many regions [24,25]. The multiple anthropogenic stressors to which they are exposed, as well as their slow population dynamics (slow growth rates, scarce recruitment, longevity; e.g. [26]) and their limited population connectivity [27], have been argued to be the main causes of their decline [18,26,28]. Nevertheless, little is known about their dispersal abilities and population dynamics, especially for deep-water macroalgal species.

*Cystoseira* species are often assumed to present low dispersal distances [28], given that their zygotes develop closely attached to the thallus of adult stands, although quantitative data regarding this topic are still scant (but see [16,29,30]). Genetic tools have brought the opportunity to unravel large-scale connectivity patterns, but there are very few genetic studies dealing with *Cystoseira* species (but see [27,31]). In contrast to genetic studies, classical experiments have provided important insights in short- to medium-distance dispersal for other algae and terrestrial plant species [13,32]. Thus, when no genetic markers are available, experimental studies can provide useful information about the effective dispersal of species [13].

Here we have experimentally determined the effective dispersal at small-scale of *Cystoseira zosteroides* C. Agradh (Fucales, Ocrophyta), a canopy-forming macroalga, which thrives in mesophotic Mediterranean rocky bottoms. Given previous studies on *Cystoseira* species, we expected that *C*. *zosteroides* would exhibit limited dispersal ability. Density-dependent survival of recruits was also expected because of the intraspecific competition with adults [33]. Finally, in order to put our results in a broader context, we compared our data with similar experiments about brown macroalgae dispersal reported in the literature. The final aim of this study was to better understand the dispersal and recruitment dynamics of macroalgae, discussing how important these outcomes are for conservation actions and habitat restoration plans.

## Material and methods

### Study species

*Cystoseria zosteroides* is an endemic and one of the most representative species of Mediterranean deep-water *Cystoseira* assemblages, inhabiting in rocky bottoms exposed to unidirectional currents at depths ranging from 15 to 50 m, with light levels ranging from 1% to 0.3% of surface irradiance [23,34,35]. Their distribution spans all the western Mediterranean Sea with the exception of the Alboran Sean [36]; however, recent studies suggest that their distribution is increasingly fragmented, mainly due to human stressors [25]. It is a long-lived species (about 50 years) and is very vulnerable to disturbances [23,26,37]. *C*. *zosteroides* has a perennial thallus with reservoir vesicles (tophules) at the top. Tophules are formed annually and constitute the origin of primary branches, which emerge during the productive season (early spring) until late summer or early autumn [37]. *C*. *zosteroides* is considered monoecious with a diplontic and iteroparous life cycle. Gametes are enveloped on parenchymatic walls which form “small bags” named conceptacles, which tend to aggregate into groups of 5 to 8 conceptacles on the basis of primary deciduous branches named receptacles. Their reproduction starts between late March and early April, and lasts until late June-early July (pers. obs.). The liberation of male gametes results in the external fertilization of female gametes, which remain attached to the external part of the receptacles. The age of sexual maturity is reached at three years (P. Capdevila unpublished data). There is no planktonic stage, zygotes are non-motile, and this species does not present clonal reproduction.

### Study site and experimental design

**Fig 1:**
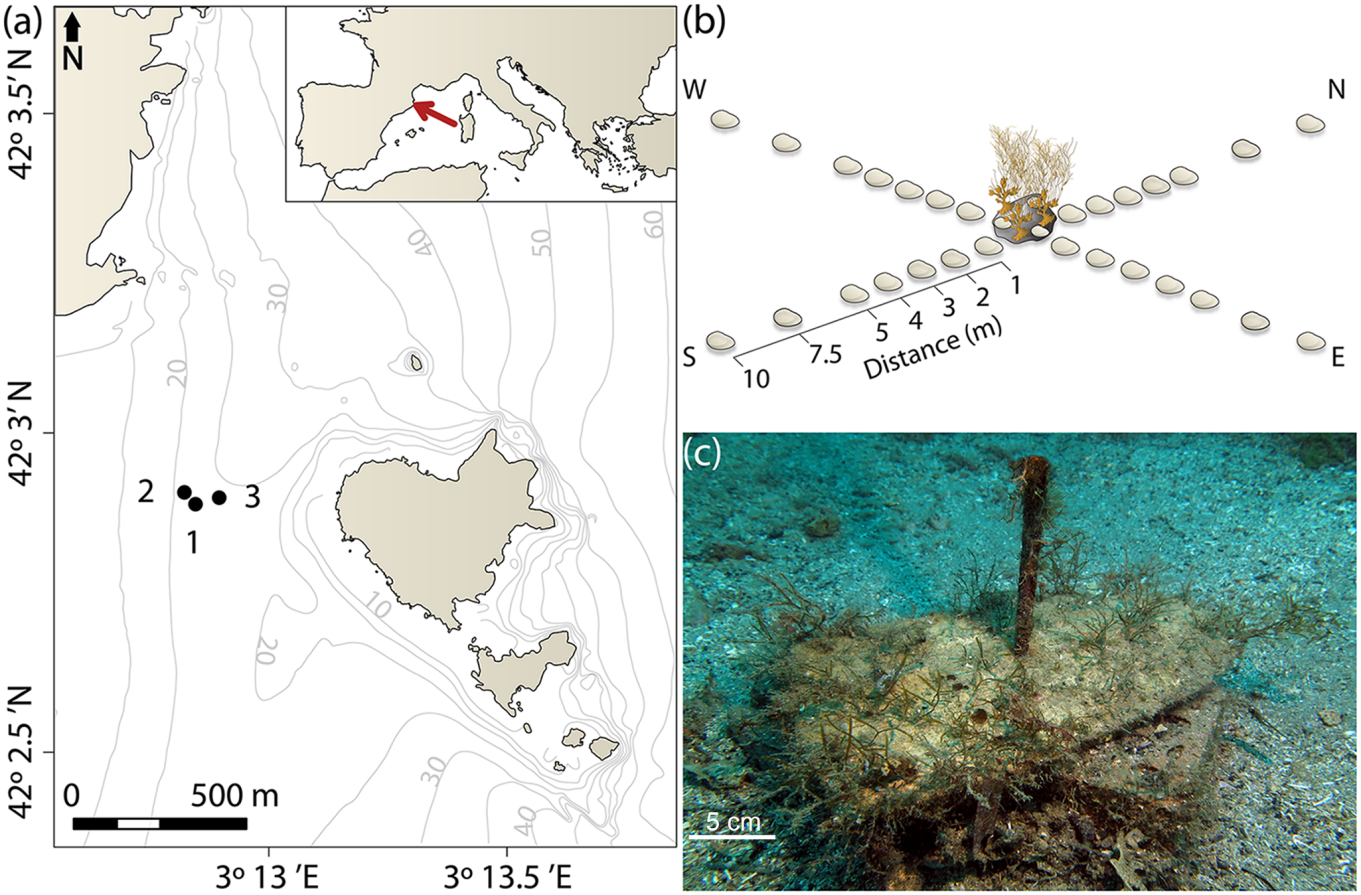
Experimental design. (a). Map of the study area. Black dots represent the sites where the experiments were installed. Map was created using R software [44]. (b). Scheme of the experimental design, with a population of *C*. *zosteroides* surrounded by sand and collectors placed at different distances (from 0 to 10 m). (c). Picture of a recruit collector after one year of being installed. The topographic base map (1:5.000), coastal limit and bathymetry are freely accessible through the Cartographic and Geologic Institute of Catalonia (www.icgc.cat) under Creative Commons Attribution License (CC BY 4.0).

Experiments were conducted at three well-developed *C*. *zosteroides* populations on Montgrí, Illes Medes and Baix Ter Natural Park (Catalonia, Spain; Site 1: N 42° 2’ 53.272” E 3°12’ 50.943”; Site 2: N 42° 2’ 54.38” E 3° 12’ 49.546”; Site 3: N 42° 2’ 53.873” E 3° 12’ 53.928”; Fig 1a). These populations were selected because they showed densities (2–4 individuals/0.25m^2^) and size structures (dominated by mature individuals) similar to other mature populations studied at the same location and with a similar extension [38]. All the three populations were found on isolated rocks surrounded by sand at a depth range of 20–28 m, separated between 50 and 100 meters from each other. For this reason, we assumed that the exchange of individuals among these populations and during the period of study was unlikely, given the limited dispersal of *Cystoseira* species [28]. The sandy bottoms surrounding these populations provided the ideal conditions to deploy this dispersal experiment, because of the isolation of the populations studied (at the short-term) and thus, facilitating the identification of the propagule source.

The particular conditions of our studied populations enabled us to place four sets of recruitment collectors on the surrounding sandy bottom at a distance of 1, 2, 3, 4, 5, 7.5 and 10 m from the edge of the *C*. *zosteroides* populations, following a cross in N, E, S and W directions (Fig 1b). In addition, three additional collectors were located inside each population, which were considered as distance 0 (Fig 1b). This resulted in a total of 31 collectors placed at each site. Collectors were made by irregular calcareous tiles, with the same composition as the rocks of the area to simulate the natural recruitment substrate of this species (Fig 1c). Inside the populations, recruitment collectors were fixed to the bottom by epoxy putty; while outside the populations, we used a metal bar crossing a drilled hole in the center of the tile and nailed into the sand to immobilize them. To prevent collectors from being buried by sand, they were laid on a brick keeping them about 15 cm above the bottom (Fig 1c). The area of each tile was estimated by photography, comparing the number of pixels occupied by the tile with a reference surface of the known area, using the software Adobe Photoshop version CS5 (Adobe Systems, San Jose, California).

Collectors were placed at Site 1 in February 2014 and the experiment was replicated in February 2015 at Sites 2 and 3, before the reproductive period of *C*. *zosteroides*. In preliminary studies, we observed that the reproductive period of this species spans from the beginning of April to late June and recruits were visually distinguishable in July. For this reason, we first estimated recruitment abundance at all collectors in July and after 60, 120 and 365 days to improve the survival estimates, although for the density-dependence analyses we only considered the annual survival. The number of individuals was estimated *in situ* by visual census of the same observers. In Site 1 they were counted during 2014–2015 and in Sites 2 and 3 were counted during 2015–2016.

### Ethics statement

Permission to work in Medes Islands was granted by the Montgrí, Illes Medes and Baix Ter Natural Park Staff. This study did not involve the removal or harvesting of *C*. *zosteroides*, which is listed in the Bern Convention and in the Aspim Protocol.

### Dispersal kernel fitting

Dispersal kernels represent the statistical distributions of dispersal distances in a population [39], or the probability density functions describing the distribution of the post-dispersal locations from a source point. To estimate the dispersal ability of *C*. *zosteroides*, we fitted seven commonly used dispersal models [39,40] to the field data: (i) power exponential, (ii) negative exponential, (iii) Gaussian, (iv) inverse Gaussian, (v) log-normal, (vi) Weibull and (vii) 2Dt (Table 1). The functions selected here are dispersal location kernels [39], projected in a two-dimensional space, so they consider the increasing area of the circle when increasing distance from source [39,40]. Then, we fitted the empirical models expressed in term of counts rather than densities [41,42]. We multiplied these functions by another fitted parameter *Q*, which represents the number of propagules dispersed, and *A*, the total area sampled by the recruit collectors at distance *r*. Then, the recruit counts (*c*) were estimated as:

c=AQf(r) (1)

**Table 1:** Dispersal functions fitted to the four cardinal directions (N,S, W and E) obtained from [39,40]. In all cases, *a* is a scale parameter, *b* is a shape parameter and *r* is the radial distance from the recruit source.

Function	Equation
2Dt	$\frac{\left(b-1\right)}{\pi a^{2}}(1+\frac{r^{2}}{a^{2}})$
Negative Exponential	$\frac{1}{2\pi a^{2}}exp(-\frac{r}{a})$
Power Exponential	$\frac{1b}{2\pi a^{2}\Gamma (\frac{2}{b})}exp(-(\frac{r^{b}}{a^{b}}))$
Gaussian	$\frac{1}{\pi a^{2}}exp(-\frac{r^{2}}{a^{2}})$
Inverse Gaussian	$\frac{\sqrt{b}}{\sqrt{8\pi^{3}r^{2}}}exp(-\frac{b{\left(r-a\right)}^{2}}{2a^{2}r})$
Log-normal	1(2π)32br2exp(-log(ra)22b2)
Weibull	$\frac{b}{2\pi a^{b}}r^{b-2}exp(-{\left(\frac{r}{a}\right)}^{b})$

The respective parameters of the dispersal kernels in Table 1 and *Q* were fitted to our data by a maximum-likelihood approach using the function “mle2” in the package “bbmle” [43] of the R software [44]. To evaluate goodness-of-fit of the models and select the best-fitted models to our data we used the Akaike Information Criterion (AIC [45]):

$${AIC}_{i}\;=\;-2\;\text{log}\;L_{i}+2V_{i}$$ (2)

where *L*_*i*_, the maximum likelihood for the candidate model *i*, is determined by adjusting the *V*_*i*_ free parameters in such a way as to maximize the probability that the candidate model has generated the observed data. To select the best candidate model, we also computed the differences in AIC, with respect to the best candidate model (with the minimum AIC value) [46]:

$$\Delta {AIC}_{i}\;=\;{AIC}_{i}-min(AIC)$$ (3)

Finally, to estimate the probability that the selected model was the best one given our data and the set of candidate models, we estimated the Akaike weights *w*_*i*_(AIC) [46]:

$$w_{i}(AIC)\;=\;\frac{exp(-\frac{1}{2}\Delta ({AIC}_{i}))}{\sum_{k\;=\;1}^{K}exp(-\frac{1}{2}\Delta ({AIC}_{k}))}$$ (4)

where K is the number of fitted models.

Dispersal studies usually assume that dispersal is isotropic (i.e. is the same in all directions [47]), so they usually don’t split the data according to the direction (but see [41]). Here, given that *C*. *zosteroides* usually inhabits deep rocky bottoms exposed to unidirectional currents [23,35,37], to account for a potential anisotropy derived from dominant currents [12,48] we fitted individual models, splitting the data by direction and site. Once we obtained the models, we selected the most frequent best-fitting function across sites and directions [47]. This enabled us to find the most representative dispersal kernel for our studied populations at a fine scale. In addition, dispersal distances estimated from different models depend much on the selected models and on the dispersal system [49]. For this reason, to accurately predict and compare the dispersal distances between sites and directions we selected the best-fitting function as a general description of our system. Finally, at each direction and site, we estimated the mean and the tail of the dispersal distance as the median and the 95^th^ percentile distances (m) respectively, formulated as the integral of the best-fitted dispersal kernel [39].

### Recruit survival

Recruit survival was estimated as the difference in the abundance between censuses, considering the starting point when recruits were visible to the unaided eye (July), which almost coincides with the end of the reproductive season. To test for differences in recruit survival between sites and time (fixed factor), we applied generalized linear mixed models (GLMM), with a binomial error distribution and a logit link function, using the ID of each tile as a random factor. We applied a Type II Wald χ^2^ test over the fitted model to determine the effect of site and time on recruit survival. GLMMs were used to deal with the non-independence between observations (i.e. repeated measures) and a binomial distribution was assumed to deal with the dichotomous response variable (survive or not survive).

We tested for differences in yearly recruit survival inside and outside the *C*. *zosteroides* canopy. To compensate for the unbalanced design of the number of recruitment plates inside and outside the canopy we only used the dispersed individuals at the 0 m and 1 m distance. We fitted a generalized linear model (GLM), with a binomial error distribution and a logit link function. To test the null hypothesis of no effect of the scaling parameter in both models (sites and inside/outside canopy respectively) we used a Wald χ^2^ test. All analyses were performed using the package “lme4” [50] of the R software [44]. The normality of residuals and overall model performance was visually inspected by observing the residual distributions and quantile-quantile plots (Figures A and B in S1 File).

### Comparative study

In order to put our results in a broader context, we compared our data with other experimental studies targeting brown macroalgae. Dispersal estimates for macroalgal species were obtained from two published reviews [8,21] and our own literature survey. A total of 42 studies were found but only direct or experimental estimates measuring the settlement or recruitment at different distances from a propagule source were used, leading a total 17 studies. We did not consider genetic studies in order to avoid potential biases due to different methodologies and approaches. Due to the scarcity of data for several macroalgae orders, comparisons were performed only between the orders Laminariales and Fucales, which were the ones with the highest number of studies (7 and 10, respectively).

## Results

### Dispersal distances

A total of 727, 791 and 494 recruits were found at Sites 1, 2 and 3 respectively at the first census. Although some recruit collectors yielded no recruits, when considering all together, recruits were found at all distances and directions. For the Site 3 the directions North and East contained very low recruit densities, so we did not fit the individual distributions to avoid misleading dispersal estimates. At all sites, 95% of recruits fell within 7.5 m from the algal stand edge. The zygote abundance at 0 m was very variable.

**Fig 2:**
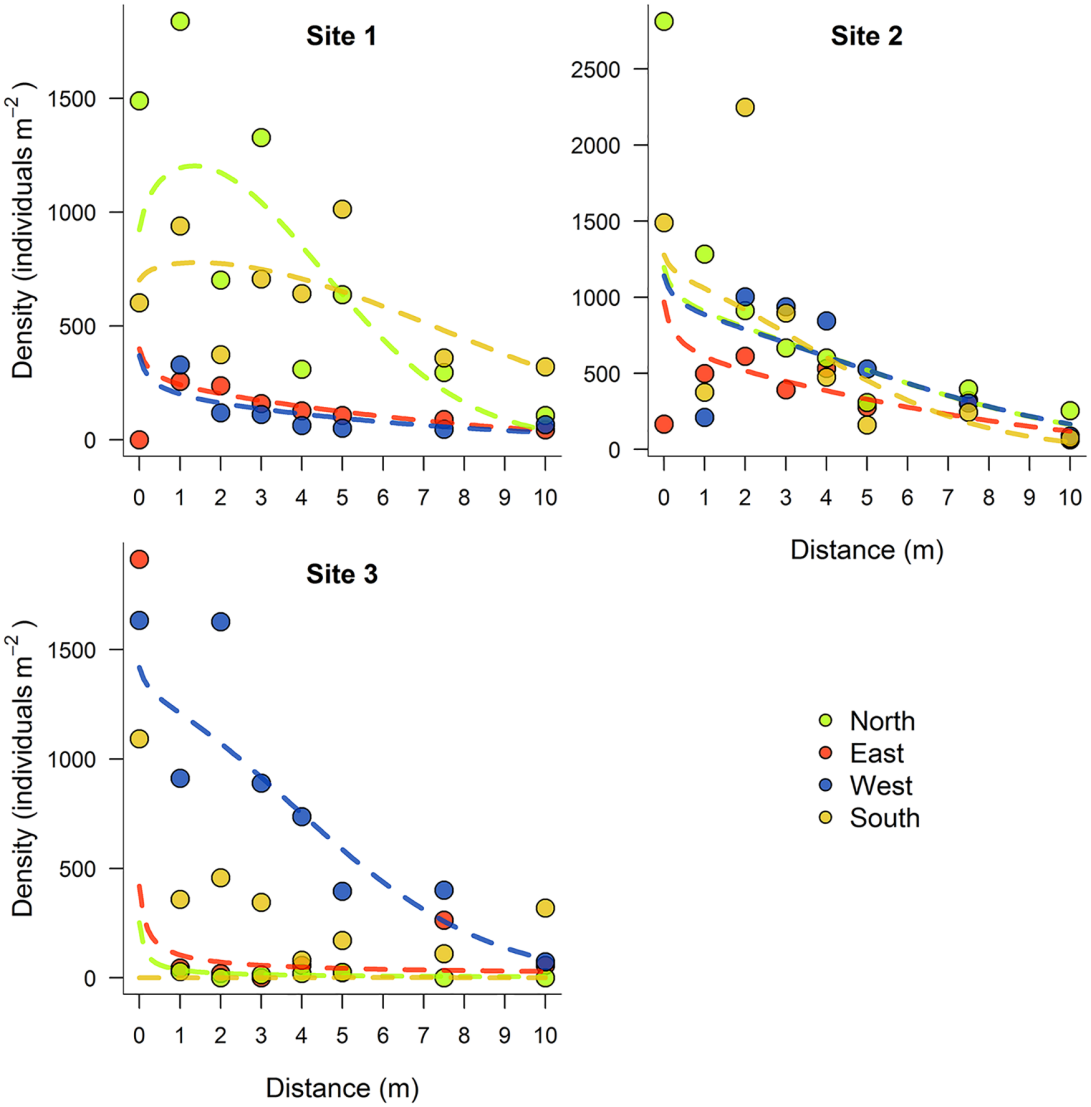
Density of *Cystoseira zosteroides* recruits at different distances from the source population. Lines indicate the predictions from Weibull model fit to the data. Panels contain the different studied sites, and colors indicate the different orientations.

The shape of the fitted dispersal kernels for all *C*. *zosteroides* populations and directions suggests a leptokurtic distribution, with many propagules deposited close to the source and a rapid decline in recruit densities with increasing distances (Fig 2). Of the 7 functions used to model the dispersal with distance, the Weibull function generally provided the best fit for all four cardinal directions and the three sites (Tables A-C in S1 File). The median and 95^th^ quantile dispersal distances calculated from the Weibull dispersal kernels were very similar among sites and directions (Table 2). The 50^th^ quantile of the dispersion values ranged from 4.65 to 8.31 m, with a mean value of 6.43 m, while the 95^th^ quantile values ranged from 8.61 to 16.39 m, with a mean value of 13.33 m (Table 2).

**Table 2:** Fitted maximum-likelihood Weibull function values of log(Q), *a* and *b*, for each site and orientation. All the parameters showed were significantly different to 0, see Table A in S1 File. In addition, the median and 95th dispersal distances (in meters) are presented as measures of the average dispersal distance and its tail, respectively.

Site	Orientation	Log(Q)	*a*	*b*	Median	95th
1	E	10.654	8.563	1.802	6.99	15.76
1	N	11.816	5.520	2.121	4.65	9.27
1	W	10.398	8.560	1.756	6.95	16.00
1	S	12.480	9.936	2.046	8.31	16.99
2	E	11.680	8.954	1.817	7.32	16.39
2	N	12.016	8.162	1.897	6.73	14.58
2	W	12.020	8.208	1.904	6.77	14.62
2	S	11.576	5.751	1.940	4.76	10.14
3	E	-	-	-	-	-
3	N	-	-	-	-	-
3	W	11.870	6.229	1.948	5.16	10.95
3	S	5.469	7.050	5.491	6.59	8.61

### First year of survival and density-dependence

**Fig 3:**
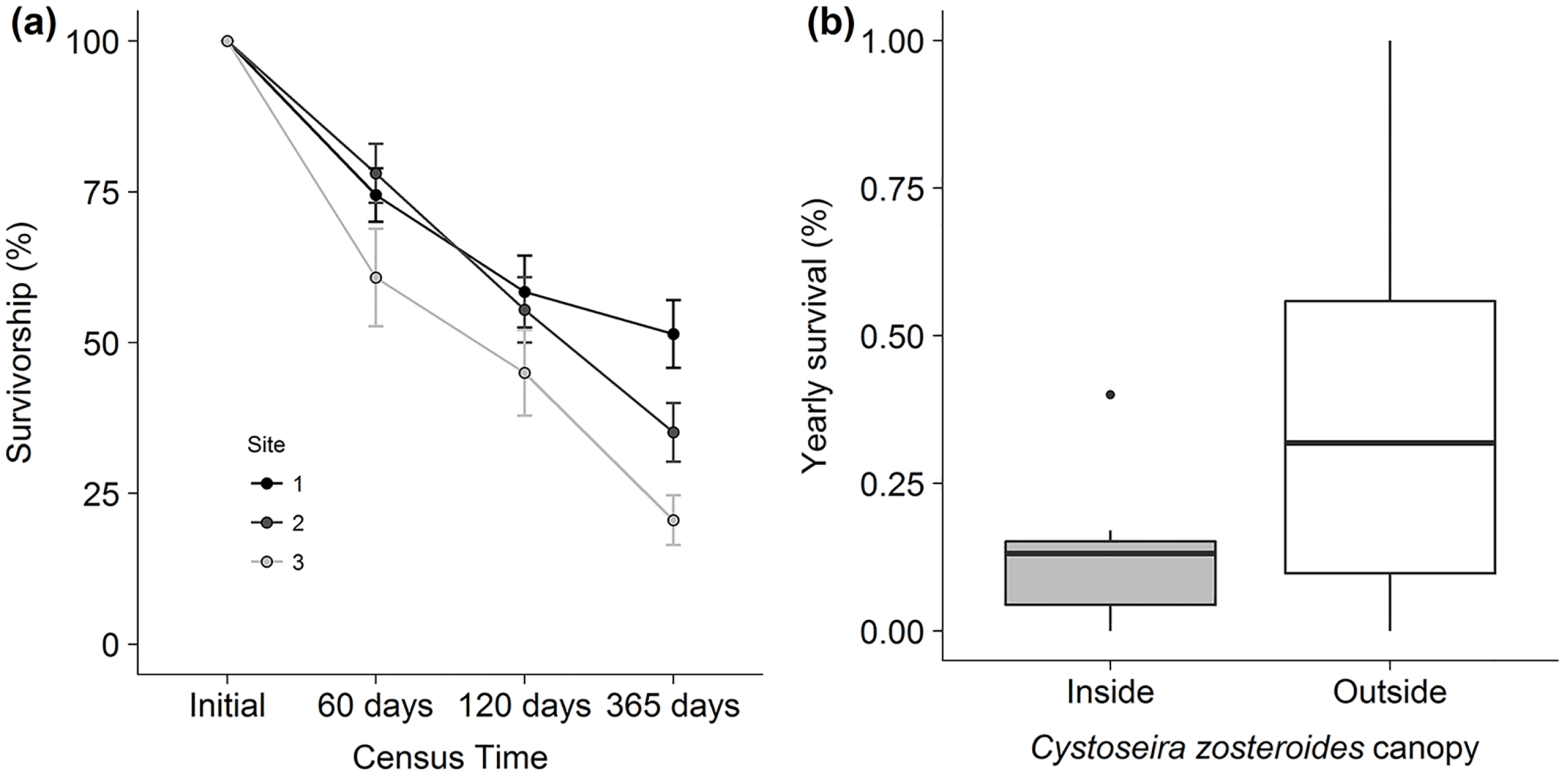
Recruit survival over the experimental deploiment. (a) Recruit survival (mean ± SE) vs census time. Grey scale indicates the different populations studied. (b) Yearly recruit survival inside and outside the *Cystoseira zosteroides* canopy. Boxes represent the interquartile range, the horizontal line represents the median, vertical line represents the upper and lower extreme values.

Survival since the first census decreased substantially with time, reaching values lower than 50% after a year (Fig 3a). Significant differences were found in recruit survival among the three populations studied (χ^2^ = 17.73, df = 2, P < 0.01) and between the consecutive years (χ^2^ = 753.52, df = 2, P < 0.01), with a significant interaction between the aforementioned factors (χ^2^ = 7.82, df = 4, P = 0.02).

In agreement with our expectations, we found that recruit survival was significantly lower inside than outside the *C*. *zosteroides* canopy (χ^2^ = 107.35, df = 1, P < 0.01; Fig 3b), indicating negative density-dependence on post-settlement survival. There were significant differences in the survival between sites (χ^2^ = 8.37, df = 2, P < 0.05) and the interaction between factors was significant (χ^2^ = 6.12, df = 2, P < 0.05).

### Comparative analyses

*Cystoseira zosteroides* was the fucoid species with the highest mean dispersal estimates (Table 3). The maximum reported mean dispersal distance was for *Pterygophora californica*, dispersing as far as 500 m (Table 3). However, another kelp species displayed the lowest dispersal distance reported, *Undaria pinnatifida* dispersing only 17.5 cm. On the other hand, the maximum mean dispersal reported for a fucoid species apart from *C*. *zosteroides* was *Sargassum horneri* (6.3 m), while *C*. *compressa* and *C*. *amantacea* showed the lowest, only 20 cm (Table 3).

**Table 3:** Results of the literature survey on the dispersal distances of macroalgae species. The mean dispersal distance is reported (in m) as well as the order of the species, the source reference and the quantification method of the dispersal distance (propagule settlement or recruitment).

Species name	Order	Method	Mean dispersal (m)	Reference
***Alaria esculenta***	Laminariales	settlement	10.00	[21]
***Fucus distichus***	Fucales	settlement	1.00	[73]
***Laminaria hyperborean***	Laminariales	settlement	200.00	[74]
***Macrocystis pyrifera***	Laminariales	settlement	25.00	[75]
***Pterygophora californica***	Laminariales	settlement	500.00	[75]
***Postelsia palmaeformis***	Laminariales	settlement	3.00	[76]
***Sargassum muticum***	Fucales	recruitment	3.00	[77]
***Sargassum polyceratium***	Fucales	recruitment	1.00	in [78]
***Cystoseira compressa***	Fucales	recruitment	0.02	[29]
***Cystoseira amentacea***	Fucales	recruitment	0.02	[29]
***Cystoseira zosteroides***	Fucales	recruitment	6.42	this study
***Sargassum spinuligerum***	Fucales	settlement	0.25	[79]
***Sargassum*** spp.	Fucales	settlement	0.25	[30]
***Ascophyllum nodosum***	Fucales	recruitment	1.00	[32]
***Undaria pinnatifida***	Laminariales	settlement	0.02	[80]
***Lessonia spicate***	Laminariales	settlement	0.50	[81]
***Sargassum horneri***	Fucales	settlement	6.30	[82]

## Discussion

Few studies have examined effective dispersal of macroalgal species at small spatial scales [21,32] and very few have fitted empirical dispersal kernels [13]. Our results suggest that *C*. *zosteroides* effective recruitment is limited to the vicinity of their populations, corroborating the limited connectivity of *Cystoseira* populations [27,31]. The recruit abundance declined with distance from the source populations, with a sharp decrease beyond the first meters, following a Weibull distribution. This dispersal kernel is commonly used in terrestrial ecology [39,51], although is usually outperformed by other fat-tailed kernels such as the log-normal or the 2Dt [39]. Still, comparing it with other fucoid species, *C*. *zosteroides* shows the highest mean dispersal reported.

Estimates of the median and the tail of the dispersal were very similar among sites and directions. Currents and waves can extend the dispersal of macroalgae propagules far from population sources [13], so given that *C*. *zosteroides* usually inhabits in rocky bottoms exposed to unidirectional currents [35,37], we expected higher dispersal distances in some directions, showing anisotropy in the movement of propagules. Nevertheless, the influence of major currents is difficult to detect with experimental approaches, which usually underestimate large-scale dispersal [4]. Genetic [48] or experimental studies including larger scales and *in situ* physical measures [13] are more likely to be able to detect directionality on the propagules dispersal. Furthermore, dispersal patterns were quite variable among the three studied sites, supporting the notion that dispersal variability tends to be large at very small spatial scales [32].

*Cystosiera zosteroides* recruit survival was low, in line with other long-lived macroalgal [52–54] and sessile species, both marine [55,56] and terrestrial [57]. *C*. *zosteroides* recruits displayed a high mortality (>50%), which contrasts with the high survival of adults (more than 90% [23]). It is worth noting that here we missed a substantial proportion of this mortality, which happens during settlement phases when individuals are not visually distinguishable [16]. Besides, recruit survival was site-dependent, supporting the high variability at small spatial scales observed in *C*. *zosteroides* population dynamics studied elsewhere [35,38]. These results suggest that a deeper comprehension of the environmental factors driving recruit survival is still needed for this species.

Although recruitment was high inside *C*. *zosteroides* populations, recruit survival was lower than outside the adult canopy [23,33,38]. These results show that adult canopy exerts a negative influence on the recruit survival, yet the underlying mechanisms behind this negative density-dependence cannot be revealed with the data presented here. However, given the depth at which *C*. *zosteroides* dwell, light inhibition by conspecifics has been suggested to be a major restricting factor [33]. Indeed, laboratory studies show that light limitation can diminish growth and survival rates of early macroalgal stages [58,59]. On the other hand, propagules arriving in high densities at natural gaps can experience higher mortalities than those distributed sparsely over larger areas [60,61]. This suggests that given the higher densities of recruits inside the populations, early competition could lower the survival of recruits. Although with the present data we cannot confirm this hypothesis, our results support that *C*. *zosteroides* do have a strong ability to compensate disturbance pulses through the arrival of recruits to cleared zones [26,33]. Further, the negative density-dependence does explain the recruitment limitation observed in natural *C*. *zosteroides* populations [23,33] and it is likely in many other subtidal macroalgae [16].

Limited dispersal and low early survival seem to be common traits among brown habitat-forming macroalgae species [16,21]. Compared to other macroalgal species, *C*. *zosteroides* displays a dispersal range similar to most fucoids, but lower than most kelps. The decline of *Cystoseira* species across the Mediterranean has been argued to be a consequence of the paucity of their populations and their limited dispersal abilities [25]. Supporting these studies, we show that *C*. *zosteroides* effective dispersal is very limited, also in line with the high genetic differentiation observed among other *Cystoseira* species [27,31]. Sporadic dispersal events through drifting thalli or dislocated fertile algae have been suggested to enhance connectivity among distant *Cystoseira* populations [27]. Yet, these seem very unlikely in our case, given the lack of air bladders for *C*. *zosteroides* and the low survival of recruits, suggesting that these rare events may not be enough to recover entire extensions of their populations at short-time scales, as seen in other macroalgae species [62]. For our studied species, which inhabits in relatively stable habitats [37] and present slow population dynamics [26], short-dispersal could be a mechanism to maintain local populations over more competitive species. Although this local retention of propagules intensifies intraspecific competition, through negative density-dependence, it acts as a reservoir of recruits to compensate disturbance pulses [26,33,38]. Nevertheless, the low recruit survival and the low dispersal ability of *C*. *zosteroides* highlights their poor ability to colonize new habitats and respond to large disturbances affecting broad extensions [23,38]. This, coupled with the late age at maturation, the slow somatic growth and the patchiness of *C*. *zosteroides* populations could arguably explain local extinctions [25,26,63].

The patchy distribution of *C*. *zosteroides* populations across the Mediterranean and the uncertainty of their distribution in several regions, coupled with the lack of genetic markers, prevented us from studying the connectivity of this species at a larger temporal and spatial scales. Furthermore, their deep distribution challenges more exhaustive studies that could help us to clear up the underlying dispersal mechanisms. Nevertheless, our approach enabled us to have an estimate of their short-scale dispersal, as well as their survival, during their macroscopic recruit stage. The limited dispersal and recovery ability of other fucoid species [64,65] strengthens the patterns observed here. For this reason, our study constitutes the first step in understanding the dispersal mechanisms of this species, but future studies should consider incorporating broader spatial and temporal scales.

Overall, our results illustrate that the effective dispersal of the deep-water macroalga *C*. *zosteroides*, similarly to other fucoid species, is limited to few meters. Although *C*. *zosteroides* is the fucoid species with the highest propagule dispersal ability, their effective dispersal is still much lower than for other marine species [21,22]. These results indicate that their loss in fragmented areas could be hardly reversible, given that the limited dispersal and the paucity of their populations would prevent rapid recolonization and would extend the recovery time. Indeed, the recovery of *C*. *zosteroides* populations can take decades [26]. This contrasts with many ephemeral kelp forests, which can be eliminated within a year but are able to recover as quickly as they have disappeared [66,67]. The differences in the dispersal patterns, as well as contrasting population dynamics [26], may explain divergences in the recovery dynamics of macroalgae species, suggesting that dispersal scales must be taken into account when designing management plans for these and other marine species [7]. In the case of our studied *C*. *zosteroides* populations, they may be favoured by increasing their protection against human stressors (e.g. pollution, physical disturbances), but their recovery may need restoration actions, such as adult transplantation [68] or seeding techniques [69]. This could be the case of other fucoid species with limited dispersal and low recovery abilities. Given the global decline of macroalgal forests [24,70–72], ensuring the connectivity of their populations should be a conservation priority in temperate seas, but we highlight here that this requires a clear comprehension of the dispersal mechanisms and the life history of targeted species.

## Supporting information

S1 File Complementary analyses. Model diagnostics (Figures A and B), individual (for each direction and site) dispersal models (Table A), AIC results for individual models (Table B) and best-fitted models summary (Table C).
(PDF)
