# Supplementary material for: Effective dispersal and density-dependence in mesophotic macroalgal forests: Insights from the Mediterranean species Cystoseira zosteroides
Source: PLoS One. 2018 Jan 12;13(1):e0191346. doi: 10.1371/journal.pone.0191346 (PMC5766243; doi:10.1371/journal.pone.0191346)
Supplement: S1 File — Model diagnostics (Figures A and B), individual (for each direction and site) dispersal models (Table A), AIC results for individual models (Table B) and best-fitted models summary (Table C). (PDF) [file pone.0191346.s001.pdf]

Supporting Information

Effective dispersal and density-dependence in mesophotic macroalgal forests: insights from the Mediterranean species *Cystoseira zosteroides*.

Pol Capdevila[1*], Cristina Linares[1], Eneko Aspillaga[1], Joan Lluís Riera[1], Bernat Hereu[1]

[1] Departament de Biologia Evolutiva, Ecologia i Ciències Ambientals, Facultat de Biologia, Universitat de Barcelona, Avda Diagonal 643, 08028 Barcelona, Spain.

*Contact author: pcapdevila.pc@gmail.com

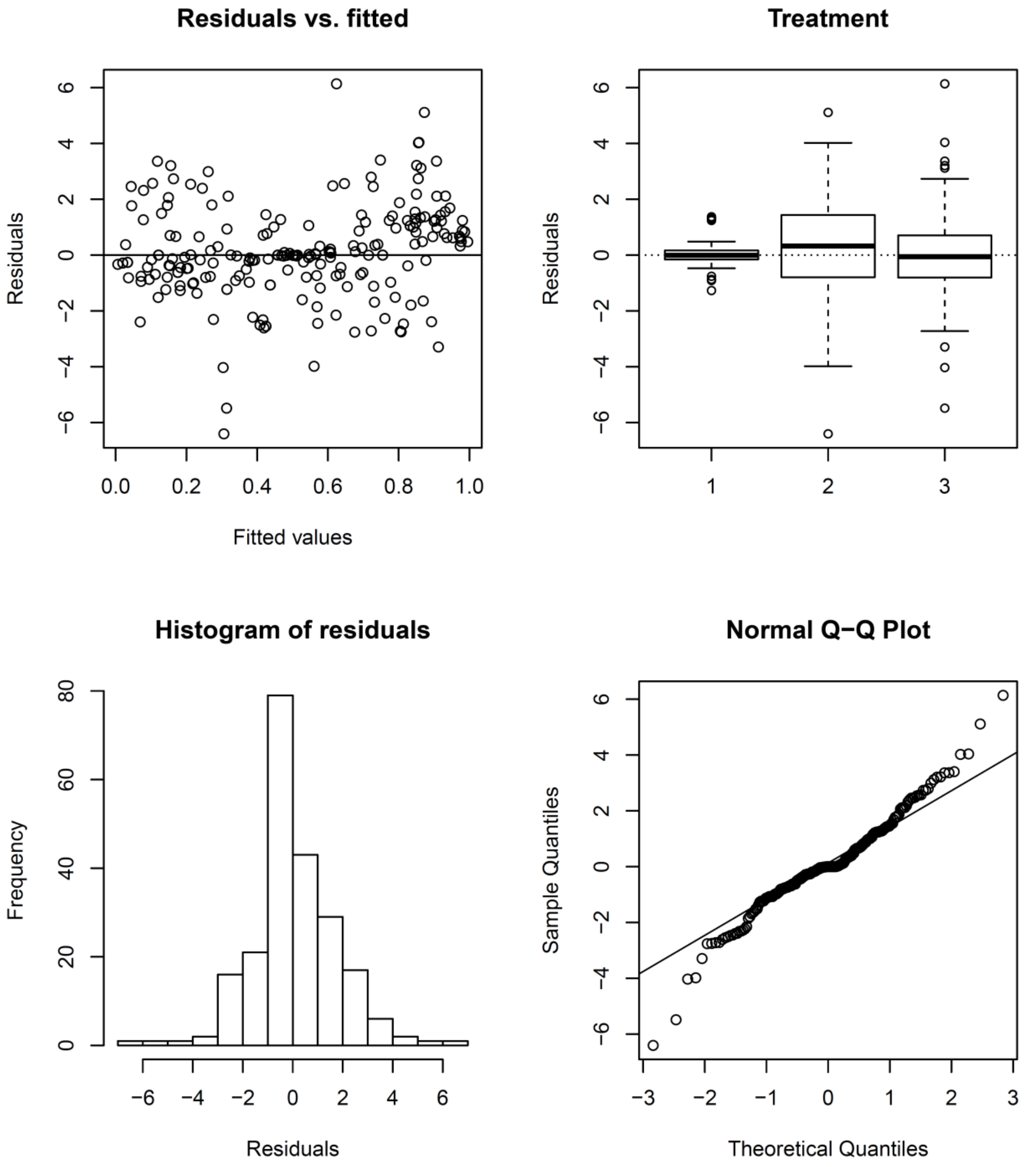

Figure A. Diagnostics of the generalized linear mixed model, with a binomial distribution and a logit link function, for recruit survival between sites and time (fixed factors) using plate as a random factor.

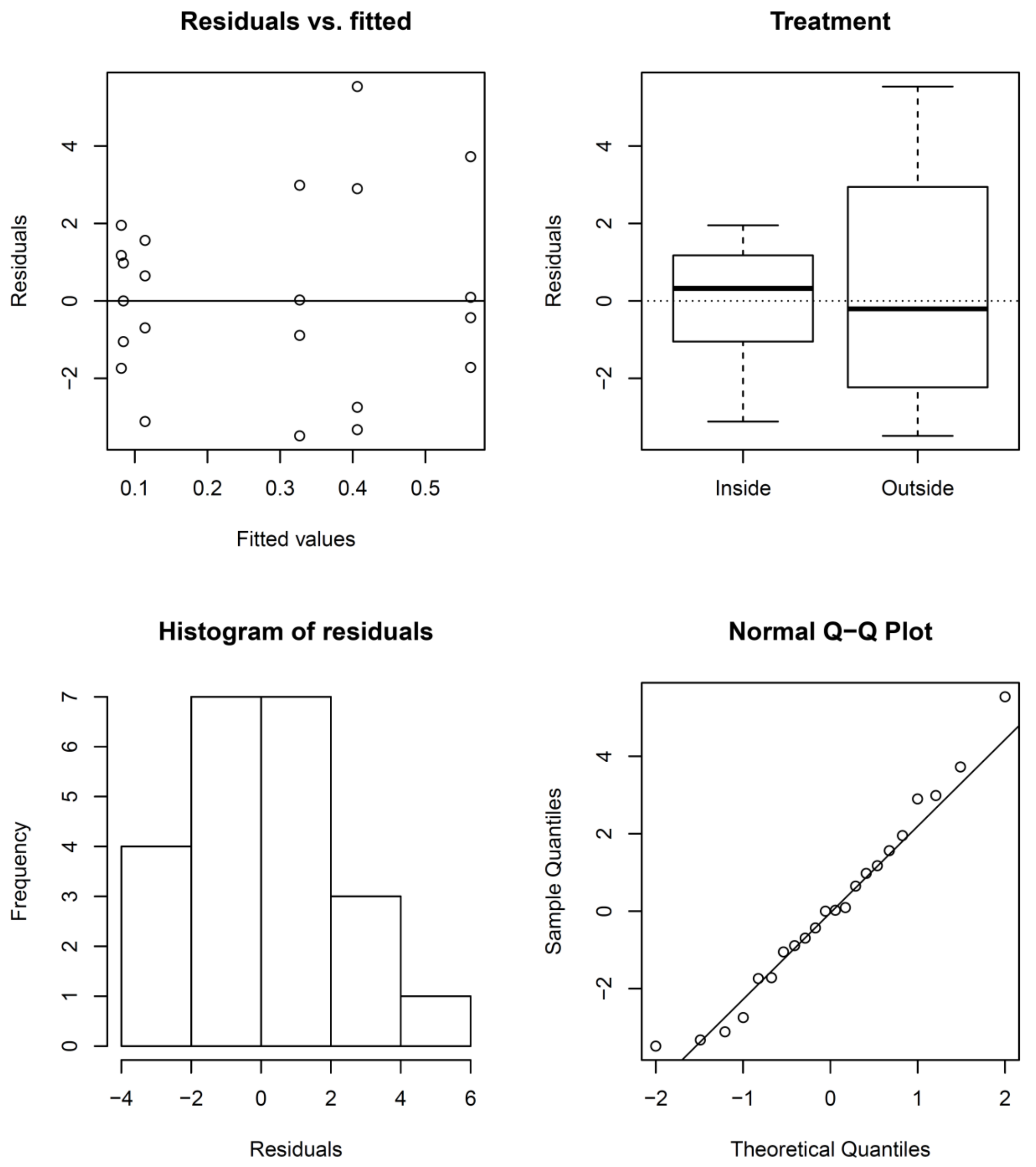

Figure B. Diagnostics of the generalized linear model, with a binomial distribution and a logit link function, for yearly recruit survival between inside and outside the adult canopy.

Table A. Maximum-likelihood estimates of all the models fitted to the data, for each site and orientation. The *a* and *b* values are the scale and shape parameters respectively, for each of the functions. Log(Q) is also a fitted parameter, which represents the logarithm of the number of propagules liberated for each direction.

Site	Orientation	Function	Parameters	z-value	p-value
1	E	2Dt	**a = 4.364**	**$3.639 \cdot 10^{8}$**	**<0.001**
			**$b = 0.87 \cdot 10^{3}$**	**11.486**	**<0.001**
			**log(Q)= 15.992**	**$2.147 \cdot 10^{8}$**	**<0.001**
1	E	Negative Exponential	**a = 3.025**	**6.631**	**<0.001**
			**log(Q)= 10.372**	**40.089**	**<0.001**
1	E	Power Exponential	*a = 1.596*	*2.258*	*0.024*
			b = 0.472	*2.800*	*0.005*
			**log(Q)= 10.422**	**6.906**	**<0.001**
1	E	Gaussian	**a = 4.947**	**9.557**	**<0.001**
			**log(Q)= 15.992**	**56.388**	**<0.001**
1	E	Inverse Gaussian	**a = 247.250**	**$3.639 \cdot 10^{8}$**	**<0.001**
			**b = 0.237**	**65.347**	**<0.001**
			**log(Q)= 10.269**	**74.531**	**<0.001**
1	E	Log-normal	**$a = 5.756 \cdot 10^{4}$**	**$1.548 \cdot 10^{10}$**	**<0.001**
			**b = 2.690**	**$1.090 \cdot 10^{2}$**	**<0.001**
			**log(Q)= 17.773**	**71.622**	**<0.001**
1	E	Weibull	*a = 8.563*	*3.0876*	*0.002*
			**b = 1.802**	**44.056**	**<0.001**
			**log(Q)= 10.654**	**23.357**	**<0.001**
1	N	2Dt	**a = 1957.446**	**$12.888 \cdot 10^{3}$**	**<0.001**
			**b = 44.124**	**6.583**	**<0.001**
			**log(Q)= 11.859**	**88.580**	**<0.001**
1	N	Negative Exponential	**a = 7.212**	**6.542**	**<0.001**
			**log(Q)= 12.772**	**47.742**	**<0.001**
1	N	Power Exponential	**a = 224.606**	**9628.707**	**<0.001**
			**b = 0.411**	**5.262**	**<0.001**
			**log(Q)= 23.289**	**14.606**	**<0.001**
1	N	Gaussian	**a = 6.645**	**13.410**	**<0.001**
			**log(Q)= 11.853**	**90.214**	**<0.001**
1	N	Inverse Gaussian	**a = 867.700**	**$4.143 \cdot 10^{10}$**	**<0.001**
			**b = 0.268**	**105.610**	**<0.001**
			**log(Q)= 11.742**	**199.340**	**<0.001**
1	N	Log-normal	a = 63.538	1.823	0.068
			**b = 1.701**	**21.462**	**<0.001**
			**log(Q)= 13.775**	**27.544**	**<0.001**
1	N	Weibull	**a = 5.520**	**16.343**	**<0.001**
			**b = 2.121**	**89.856**	**<0.001**
			**log(Q) = 11.816**	**118.595**	**<0.001**
1	W	2Dt	**a = 2.002**	**$3.130 \cdot 10^{8}$**	**<0.001**
			**$b = 4.437 \cdot 10^{-4}$**	**11.830**	**<0.001**
			**log(Q)= 15.940**	**$9.579 \cdot 10^{8}$**	**<0.001**

1	W	Negative Exponential	**a = 2.437**	**7.640**	**<0.001**
			**log(Q)= 9.915**	**44.931**	**<0.001**
1	W	Power Exponential	*a = 1.198*	*2.971*	*0.003*
			**b = 0.529**	**4.620**	**<0.001**
			**log(Q)= 10.818**	**14.051**	**<0.001**
1	W	Gaussian	**a = 4.825**	**10.026**	**<0.001**
			**log(Q)= 10.239**	**58.892**	**<0.001**
1	W	Inverse Gaussian	**a = 263.220**	**$6.270 \cdot 10^8$**	**<0.001**
			**b = 0.229**	**65.943**	**<0.001**
			**log(Q)= 9.866**	**77.263**	**<0.001**
1	W	Log-normal	**a = 22.597**	**$1.809 \cdot 10^5$**	**<0.001**
			**b = 2.052**	**107.190**	**<0.001**
			**log(Q)= 12.645**	**75.780**	**<0.001**
1	W	Weibull	**a = 8.560**	**3.416**	**<0.001**
			**b = 1.756**	**46.219**	**<0.001**
			**log(Q)= 10.398**	**25.329**	**<0.001**
1	S	2Dt	**a = 540.102**	**$7.151 \cdot 10^4$**	**<0.001**
			**b = 3.486**	**3.350**	**<0.001**
			**log(Q)= 12.790**	**47.214**	**<0.001**
1	S	Negative Exponential	**a = 16.628**	**3.595**	**<0.001**
			**log(Q)= 14.098**	**27.735**	**<0.001**
1	S	Power Exponential	**a = 31.835**	**$8.599 \cdot 10^3$**	**<0.001**
			**b = 0.105**	**497.160**	**<0.001**
			**log(Q)= 54.744**	**478.240**	**<0.001**
1	S	Gaussian	**a = 11.361**	**8.315**	**<0.001**
			**log(Q)= 12.609**	**58.985**	**<0.001**
1	S	Inverse Gaussian	**a = 843.340**	**$3.836 \cdot 10^{10}$**	**<0.001**
			**b = 0.263**	**103.330**	**<0.001**
			**log(Q)= 11.510**	**193.610**	**<0.001**
1	S	Log-normal	**a =5.590**	**1.823**	**<0.001**
			**b = 2.932**	**21.462**	**<0.001**
			**log(Q)= 24.662**	**151.900**	**<0.001**
1	S	Weibull	**a= 9.936**	**9.097**	**<0.001**
			**b= 2.046**	**82.523**	**<0.001**
			**log(Q) = 12.480**	**72.720**	**<0.001**
2	E	2Dt	**a = 7.710**	**$4.130 \cdot 10^8$**	**<0.001**
			**b = $0.182 \cdot 10^{-3}$**	**16.779**	**<0.001**
			**log(Q)= 16.559**	**$8.317 \cdot 10^7$**	**<0.001**
2	E	Negative Exponential	**a = 3.670**	**9.703**	**<0.001**
			**log(Q)= 11.541**	**69.808**	**<0.001**
2	E	Power Exponential	*a = 1.807*	*3.047*	*0.002*
			**b = 0.438**	**3.824**	**<0.001**
			**log(Q)= 13.693**	**9.249**	**<0.001**
2	E	Gaussian	**a = 5.717**	**14.209**	**<0.001**
			**log(Q)= 11.442**	**96.438**	**<0.001**
2	E	Inverse Gaussian	**a = 376.100**	**$2.769 \cdot 10^9$**	**<0.001**
			**b = 0.242**	**100.82**	**<0.001**

			**log(Q)= 11.368**	**142.850**	**<0.001**
2	E	Log-normal	**a = $2.782 \cdot 10^6$**	**$4.906 \cdot 10^{13}$**	**<0.001**
			**b = 3.058**	**$1.704 \cdot 10^2$**	**<0.001**
			**log(Q)= 22.181**	**$1.233 \cdot 10^2$**	**<0.001**
2	E	Weibull	**a = 8.954**	**5.511**	**<0.001**
			**b = 1.817**	**70.477**	**<0.001**
			**log(Q)= 11.680**	**45.893**	**<0.001**
2	N	2Dt	**a = 10.700**	**$7.413 \cdot 10^8$**	**<0.001**
			**b = 0.002**	**17.337**	**<0.001**
			**log(Q)= 17.705**	**$8.469 \cdot 10^7$**	**<0.001**
2	N	Negative Exponential	**a = 4.460**	**8.775**	**<0.001**
			**log(Q)= 12.111**	**63.786**	**<0.001**
2	N	Power Exponential	**a = 4.035**	**6.307**	**<0.001**
			**b = 0.788**	**5.153**	**<0.001**
			**log(Q)= 12.507**	**25.719**	**<0.001**
2	N	Gaussian	**a = 6.215**	**13.284**	**<0.001**
			**log(Q)= 11.867**	**92.536**	**<0.001**
2	N	Inverse Gaussian	**a = 508.890**	**$7.015 \cdot 10^{10}$**	**<0.001**
			**b = 0.251**	**107.330**	**<0.001**
			**log(Q)= 11.784**	**157.010**	**<0.001**
2	N	Log-normal	**a = 107.920**	**$1.257 \cdot 10^7$**	**<0.001**
			**b = 2.170**	**0.018**	**<0.001**
			**log(Q)= 15.891**	**0.015**	**<0.001**
2	N	Weibull	**a= 8.162**	**6.871**	**<0.001**
			**b= 1.897**	**77.255**	**<0.001**
			**log(Q) = 12.016**	**57.628**	**<0.001**
2	W	2Dt	**a = $4.751 \cdot 10^3$**	**$1.144 \cdot 10^4$**	**<0.001**
			**b = 112.183**	**6.327**	**<0.001**
			**log(Q)= 11.885**	**86.087**	**<0.001**
2	W	Negative Exponential	**a = 5.508**	**7.301**	**<0.001**
			**log(Q)= 12.399**	**53.378**	**<0.001**
2	W	Power Exponential	**a = 4.049**	**11.027**	**<0.001**
			**b = 0.152**	**23.924**	**<0.001**
			**log(Q)= 34.338**	**27.036**	**<0.001**
2	W	Gaussian	**a = 6.469**	**12.808**	**<0.001**
			**log(Q)= 11.875**	**87.102**	**<0.001**
2	W	Inverse Gaussian	**a = 254.420**	**$9.297 \cdot 10^8$**	**<0.001**
			**b = 0.229**	**106.760**	**<0.001**
			**log(Q)= 11.542**	**159.480**	**<0.001**
2	W	Log-normal	**a = $1.471 \cdot 10^9$**	**$1.054 \cdot 10^{19}$**	**<0.001**
			**b = 3.515**	**$1.882 \cdot 10^2$**	**<0.001**
			**log(Q)= 28.849**	**$1.299 \cdot 10^{19}$**	**<0.001**
2	W	Weibull	**a = 8.208**	**6.969**	**<0.001**
			**b = 1.904**	**77.881**	**<0.001**
			**log(Q)= 12.020**	**56.758**	**<0.001**
2	S	2Dt	a = 27.666	1.683	0.092
			b = 0.967	1.267	0.205

			**log(Q)= 11.736**	**44.245**	**<0.001**
2	S	Negative Exponential	**a = 3.760**	**10.533**	**<0.001**
			**log(Q)= 11.817**	**75.241**	**<0.001**
2	S	Power Exponential	**a = 4.389**	**9.758**	**<0.001**
			**b = 1.390**	**6.633**	**<0.001**
			**log(Q)= 11.598**	**87.606**	**<0.001**
2	S	Gaussian	**a = 5.213**	**16.527**	**<0.001**
			**log(Q)= 11.586**	**110.201**	**<0.001**
2	S	Inverse Gaussian	**a = 232.790**	**$6.754 \cdot 10^8$**	**<0.001**
			**b = 0.250**	**106.600**	**<0.001**
			**log(Q)= 11.720**	**155.760**	**<0.001**
2	S	Log-normal	**a =381.410**	**$1.749 \cdot 10^6$**	**<0.001**
			**b = 2.042**	**178.840**	**<0.001**
			**log(Q)= 14.905**	**151.410**	**<0.001**
2	S	Weibull	**a= 5.751**	**12.419**	**<0.001**
			**b= 1.940**	**81.325**	**<0.001**
			**log(Q) = 11.576**	**98.197**	**<0.001**
3	E	2Dt	**a = 0.150**	**3.505**	**<0.001**
			b = 0.017	0.853	0.393
			**log(Q)= 10.698**	**9.383**	**<0.001**
3	E	Negative Exponential	**a = 1.355**	**1.037**	**<0.001**
			**log(Q)= 9.533**	**54.458**	**<0.001**
3	E	Power Exponential	a = 0.004	1.247	0.213
			**b = 0.198**	**15.077**	**<0.001**
			**log(Q)= 13.151**	**$6.334 \cdot 10^4$**	**<0.001**
3	E	Gaussian	**a = 4.102**	**11.726**	**<0.001**
			**log(Q)= 10.547**	**70.751**	**<0.001**
3	E	Inverse Gaussian	**a = 315.910**	**$7.291 \cdot 10^8$**	**<0.001**
			**b = 0.193**	**38.738**	**<0.001**
			**log(Q)= 9.084**	**40.405**	**<0.001**
3	E	Log-normal	**a = $2.519 \cdot 10^{11}$**	**$1.404 \cdot 10^{23}$**	**<0.001**
			**b = 4.440**	**71.400**	**<0.001**
			**log(Q)= 26.543**	**41.270**	**<0.001**
3	E	Weibull	**a = $1.302 \cdot 10^3$**	**$2.624 \cdot 10^6$**	**<0.001**
			**b = 1.423**	**33.328**	**<0.001**
			**log(Q)= 16.398**	**36.082**	**<0.001**
3	N	2Dt	a = 0.062	1.825	0.068
			b = 0.037	0.501	0.616
			**log(Q)= 9.032**	**5.114**	**<0.001**
3	N	Negative Exponential	**a = 4.460**	**8.775**	**<0.001**
			**log(Q)= 12.111**	**63.786**	**<0.001**
3	N	Power Exponential	**a = 0.001**	**0.558**	**0.577**
			**b = 0.196**	**6.763**	**<0.001**
			**log(Q)= 9.916**	**$1.476 \cdot 10^4$**	**<0.001**
3	N	Gaussian	**a = 1.466**	**14.364**	**<0.001**
			**log(Q)= 9.097**	**66.333**	**<0.001**
3	N	Inverse Gaussian	**a = 49.765**	**$1.681 \cdot 10^6$**	**<0.001**
			**b = 0.177**	**19.464**	**<0.001**

			**log(Q)= 8.352**	**19.708**	**<0.001**
3	N	Log-normal	**a = 466.810**	**$5.932 \cdot 10^{7}$**	**<0.001**
			**b = 2.941**	**28.977**	**<0.001**
			**log(Q)= 11.855**	**18.606**	**<0.001**
3	N	Weibull	**a= 30.823**	**$1.593 \cdot 10^{3}$**	**<0.001**
			**b= 1.198**	**15.421**	**<0.001**
			**log(Q) = 9.448**	**17.150**	**<0.001**
3	W	2Dt	a = 117.342	NA	NA
			b = 3.706	NA	NA
			**log(Q)= 11.890**	**103.06**	**<0.001**
3	W	Negative Exponential	**a = 4.321**	**10.289**	**<0.001**
			**log(Q)= 12.180**	**73.827**	**<0.001**
3	W	Power Exponential	**a = 4.846**	**10.071**	**<0.001**
			**b = 1.367**	**6.081**	**<0.001**
			**log(Q)= 11.916**	**79.868**	**<0.001**
3	W	Gaussian	**a = 5.652**	**16.777**	**<0.001**
			**log(Q)= 11.867**	**112.965**	**<0.001**
3	W	Inverse Gaussian	**a = 312.810**	**$1.698 \cdot 10^{9}$**	**<0.001**
			**b = 0.255**	**120.990**	**<0.001**
			**log(Q)= 12.071**	**170.180**	**<0.001**
3	W	Log-normal	**a = 350.410**	**$1.576 \cdot 10^{6}$**	**<0.001**
			**b = 2.015**	**202.24**	**<0.001**
			**log(Q)= 15.094**	**165.04**	**<0.001**
3	W	Weibull	**a = 6.229**	**12.274**	**<0.001**
			**b = 1.948**	**90.152**	**<0.001**
			**log(Q)= 11.870**	**99.730**	**<0.001**
3	S	2Dt	**a = 2.148**	**$4.400 \cdot 10^{9}$**	**<0.001**
			**b = $1.540 \cdot 10^{4}$**	**16.881**	**<0.001**
			**log(Q)= 17.912**	**$1.276 \cdot 10^{10}$**	**<0.001**
3	S	Negative Exponential	**a = 2.490**	**10.208**	**<0.001**
			**log(Q)= 10.789**	**66.725**	**<0.001**
3	S	Power Exponential	a = 0.279	1.029	0.303
			b = 0.2310	*2.650*	*0.008*
			**log(Q)= 18.009**	**3.313**	**<0.001**
3	S	Gaussian	**a = 5.157**	**11.724**	**<0.001**
			**log(Q)= 11.151**	**76.234**	**<0.001**
3	S	Inverse Gaussian	**a = 280.050**	**$1.038 \cdot 10^{9}$**	**<0.001**
			**b = 0.227**	**92.058**	**<0.001**
			**log(Q)= 10.729**	**114.780**	**<0.001**
3	S	Log-normal	**a =$7.091 \cdot 10^{6}$**	**$3.048 \cdot 10^{12}$**	**<0.001**
			**b = 2.998**	**152.840**	**<0.001**
			**log(Q)= 20.197**	**103.760**	**<0.001**
3	S	Weibull	**a= 7.050**	**8.277**	**<0.001**
			**b= 5.491**	**10.923**	**<0.001**
			**log(Q) = 5.469**	**9.764**	**<0.001**

Table B. Results of AIC analysis for the seven competing dispersal models fitted by site and direction. Here are presented: the log-likelihood score (*L*); the AIC estimates; the ΔAIC_i, which is the difference between AIC_i and the minimum value [AIC_i – min(AIC)]; and the rounded Akaike weights $wAIC_i$. Models are ranked according to their goodness-of-fit, from best to worst fitted. Best-fitted models are highlighted in grey background.

Site	Orientation	Function	*L*	AIC	ΔAIC_i	$wAIC_i$
1	E	Power Exponential	-55.4	116.9	0	0.34
1	E	Weibull	-55.5	117.1	0.2	0.31
1	E	Log-normal	-55.5	117.1	0.2	0.31
1	E	Negative Exponential	-58.8	121.7	4.8	0.03
1	E	2Dt	-60	126	9.1	0.00
1	E	Gaussian	-68.9	141.8	25	<0.001
1	E	Inverse Gaussian	-90.4	186.8	69.9	<0.001
1	N	Log-normal	-77.8	161.7	0	1.00
1	N	Weibull	-90.8	187.7	26	<0.001
1	N	Gaussian	-104.6	213.2	51.5	<0.001
1	N	2Dt	-104.6	215.2	53.5	<0.001
1	N	Negative Exponential	-114.8	233.5	71.8	<0.001
1	N	Power Exponential	-135.1	276.2	114.5	<0.001
1	N	Inverse Gaussian	-217.3	440.6	278.9	<0.001
1	W	Log-normal	-57.1	120.2	0	0.74
1	W	Power Exponential	-58.3	122.6	2.3	0.23
1	W	2Dt	-61	127.9	7.7	0.02
1	W	Weibull	-61.4	128.7	8.5	0.01
1	W	Negative Exponential	-64.2	132.4	12.2	0.00
1	W	Inverse Gaussian	-80.3	166.6	46.3	<0.001
1	W	Gaussian	-84.4	172.9	52.6	<0.001
1	S	Weibull	-76	157.9	0	0.59
1	S	Gaussian	-77.7	159.4	1.5	0.28
1	S	2Dt	-77.9	161.7	3.8	0.09
1	S	Log-normal	-78.8	163.6	5.7	0.04
1	S	Negative Exponential	-80.8	165.6	7.7	0.01
1	S	Power Exponential	-105.8	217.6	59.6	<0.001
1	S	Inverse Gaussian	-402	810	652.1	<0.001
2	E	Weibull	-71.9	149.8	0	0.82

2	E	Power Exponential	-73.8	153.7	3.8	0.12
2	E	Log-normal	-74.6	155.2	5.4	0.06
2	E	Negative Exponential	-81.3	166.6	16.8	<0.001
2	E	2Dt	-86.7	179.3	29.5	<0.001
2	E	Gaussian	-98.2	200.5	50.6	<0.001
2	E	Inverse Gaussian	-202.5	411	261.2	<0.001
2	N	Log-normal	-70	146	0	0.60
2	N	Negative Exponential	-72.1	148.3	2.3	0.19
2	N	Power Exponential	-71.3	148.6	2.6	0.16
2	N	2Dt	-72.7	151.3	5.3	0.04
2	N	Weibull	-75	155.9	9.9	0.00
2	N	Gaussian	-84.7	173.4	27.4	<0.001
2	N	Inverse Gaussian	-181.2	368.3	222.3	<0.001
2	W	Weibull	-90.3	186.6	0	0.99
2	W	Negative Exponential	-97.1	198.1	11.5	0.00
2	W	Gaussian	-98.1	200.3	13.6	0.00
2	W	Power Exponential	-97.9	201.8	15.2	<0.001
2	W	2Dt	-98.1	202.3	15.6	<0.001
2	W	Log-normal	-98.4	202.7	16.1	<0.001
2	W	Inverse Gaussian	-302.4	610.8	424.2	<0.001
2	S	2Dt	-102.8	211.6	0	0.55
2	S	Weibull	-103.8	213.6	1.9	0.21
2	S	Power Exponential	-104	214	2.4	0.17
2	S	Negative Exponential	-106.1	216.2	4.5	0.06
2	S	Gaussian	-107.2	218.4	6.8	0.02
2	S	Log-normal	-115.9	237.8	26.2	<0.001
2	S	Inverse Gaussian	-242.4	490.7	279.1	<0.001
3	E	Weibull	-38.7	83.4	0	0.82
3	E	Log-normal	-40.9	87.7	4.3	0.10
3	E	Power Exponential	-41	87.9	4.5	0.09
3	E	2Dt	-64.7	135.5	52.1	<0.001
3	E	Inverse Gaussian	-76.2	158.4	75	<0.001
3	E	Negative Exponential	-107.6	219.2	135.8	<0.001
3	E	Gaussian	-175.5	354.9	271.5	<0.001

3	N	Weibull	-18.9	43.8	0	0.38
3	N	Log-normal	-19.2	44.3	0.5	0.29
3	N	Power Exponential	-19.2	44.4	0.6	0.29
3	N	2Dt	-21.3	48.5	4.7	0.04
3	N	Inverse Gaussian	-22.7	51.4	7.6	0.01
3	N	Negative Exponential	-31.8	67.5	23.7	<0.001
3	N	Gaussian	-61.9	127.9	84.1	<0.001
3	W	Weibull	-37.2	80.5	0	0.41
3	W	Power Exponential	-37.5	81.1	0.6	0.30
3	W	Negative Exponential	-39.2	82.4	1.9	0.15
3	W	2Dt	-38.8	83.7	3.2	0.08
3	W	Gaussian	-40.2	84.5	4	0.05
3	W	Log-normal	-44.9	95.7	15.2	<0.001
3	W	Inverse Gaussian	-171.1	348.3	267.8	<0.001
3	S	Power Exponential	-36.2	78.4	0	0.74
3	S	Log-normal	-37.2	80.5	2.1	0.26
3	S	2Dt	-61	128	49.6	<0.001
3	S	Negative Exponential	-63.3	130.5	52.2	<0.001
3	S	Gaussian	-106.6	217.1	138.8	<0.001
3	S	Inverse Gaussian	-120.9	247.7	169.4	<0.001
3	S	Weibull	-373.9	753.8	675.5	<0.001

Table C. Number of times each of the models was the best-fitted to the data, for each site and direction. The selection criteria was AIC, see methods and Table B.

Model	Times best-fitted
2Dt	1/12
Negative Exponential	0/12
Power Exponential	2/12
Gaussian	0/12
Inverse Gaussian	0/12
Log-normal	3/12
Weibull	6/12
